# Differentiable phylogenetics *via* hyperbolic embeddings with Dodonaphy

**DOI:** 10.1093/bioadv/vbae082

**Published:** 2024-06-19

**Authors:** Matthew Macaulay, Mathieu Fourment

**Affiliations:** Australian Institute for Microbiology & Infection, University of Technology Sydney, Ultimo, NSW 2007, Australia; Australian Institute for Microbiology & Infection, University of Technology Sydney, Ultimo, NSW 2007, Australia

## Abstract

**Motivation:**

Navigating the high dimensional space of discrete trees for phylogenetics presents a challenging problem for tree optimization. To address this, hyperbolic embeddings of trees offer a promising approach to encoding trees efficiently in continuous spaces. However, they require a differentiable tree decoder to optimize the phylogenetic likelihood. We present soft-NJ, a differentiable version of neighbour joining that enables gradient-based optimization over the space of trees.

**Results:**

We illustrate the potential for differentiable optimization over tree space for maximum likelihood inference. We then perform variational Bayesian phylogenetics by optimizing embedding distributions in hyperbolic space. We compare the performance of this approximation technique on eight benchmark datasets to state-of-the-art methods. Results indicate that, while this technique is not immune from local optima, it opens a plethora of powerful and parametrically efficient approach to phylogenetics *via* tree embeddings.

**Availability and implementation:**

Dodonaphy is freely available on the web at https://www.github.com/mattapow/dodonaphy. It includes an implementation of soft-NJ.

## 1 Introduction

Phylogenetics provides us with the evolutionary history of a set of taxa given their genetic sequences, which is usually a bifurcating tree. However, fast optimization relies on gradients, which are not well defined between discrete trees. Thus, most tree optimization techniques consider manual changes to the tree topology before optimizing the continuous parameters (branch lengths) of each tree considered (Stamatakis 2014, Minh *et al.* 2020). Knowing which of the super-exponential number of trees to manually try is a challenging task (Guindon *et al.* 2010, Ki and Terhorst 2022).

Providing a differentiable way to move between tree topologies would allow well-developed continuous optimization techniques to work in the space of phylogenetic trees. In this article, we propose a novel technique to continuously move through the space of bifurcating trees with gradients. Our approach hinges on two ideas (a) an embedding of the genetic sequences into a continuous space and (b) an algorithm we propose called soft-NJ, which passes gradients through the neighbour joining algorithm. With these preliminaries, we can embed the tip nodes of a tree in the continuous embedding space and then optimize the locations of these nodes based on the neighbour joining tree that they decode from soft-NJ.

We use hyperbolic embeddings to represent trees in a continuous manner. This is similar to embedding points in Euclidean space, where each tip node of the tree is positioned in the space with a certain location (Layer and Rhodes 2017). However, the metric between two points is modified to give a negative curvature between (as opposed to positive curvature for points on a sphere). Hyperbolic data embeddings offer low dimensional, efficient, and precise ways to embed hierarchically clustered data (Nickel and Kiela 2017, Monath *et al.* 2019, Chami *et al.* 2020a, 2020b, Peng *et al.* 2022) or tree-like data in phylogenetics (Nagano *et al.* 2019, Corso *et al.* 2021, Iuchi *et al.* 2021, Wilson 2021, Koptagel *et al.* 2022, Macaulay *et al.* 2023).

Alternative continuous tree embedding methods are high dimensional, growing significantly with increasing taxa; BHV space grows double factorially (Billera *et al.* 2001), flattenings of sequence alignments grow exponentially (Allman and Rhodes 2008), sub-flattenings increase quadratically (Sumner 2017), as with tropical space (Speyer and Sturmfels 2004). In these spaces, each point corresponds to a single tree, making them high dimensional. Additionally, they have non-differentiable boundaries between trees, making them difficult to optimize in (Dinh *et al.* 2017). Whereas with hyperbolic embeddings, each taxon has an embedding location and together the set of taxa locations decode to a tree. This keeps the embedding space low dimensional and the number of optimization parameters linear in the number of taxa.

The goal of our approach is to optimize the embedding locations with gradient-based optimization, which requires a differentiable loss function (i.e. the likelihood or unnormalized posterior probability). This is easily achieved in other applications with carefully designed loss functions. However, in phylogenetics, there are well accepted Markov models of evolution (such as GTR or JC69), which rely on having a tree structure to compute their likelihood. To maximize the likelihood by changing the embedding locations, we developed soft-NJ—a differentiable version of the neighbour joining algorithm using automatic differentiation. It allows gradients to pass from the embedding locations into a decoded tree and the likelihood function.

We implemented soft-NJ in Dodonaphy, a software for likelihood-based phylogenetics using hyperbolic space. We demonstrate this newfound ability for phylogenetic optimization with two modes of gradient-based inference: maximum likelihood (ML) and Bayesian variational inference (VI).

VI is a Bayesian technique for approximating the posterior distribution with simple and tractable distributions, as reviewed in Blei *et al.* (2017). It indirectly finds the variational distribution that minimizes the KL-divergence between the unnormalized posterior and the variational distribution. This avoids the need to compute the normalizing constant in Bayes theorem or to resort to time consuming Markov chain Monte Carlo sampling, potentially offering significant computational speed ups.

Recently, phylogenetic VI has garnered increasing attention (Zhang and Matsen 2019, Zhang 2020, Ki 2022, Koptagel *et al.* 2022) as a promising way to cope with high dimensionality inherent to Bayesian phylogenetics. Concurrently, variational approximations have extended to general manifolds, such as hyperbolic space, where the variational density sits on the manifold (Wilson and Leimeister 2018, Tran *et al.* 2021, Peng *et al.* 2022). We combine these two paradigms to perform variational Bayesian phylogenetic inference on hyperbolic manifolds.

To perform VI on the space of phylogenies, we equip each of *n* embedded taxon locations with a variational distribution (a projected multivariate-Normal) in hyperbolic space $H^{d}$. We optimize the set of *n* probability distributions in hyperbolic space. We can quickly draw samples from these distributions and compute their neighbour joining tree of the sample. This yields a distribution of phylogenetic trees that approximate the posterior distribution.

The broader implications of this work extend beyond the field of phylogenetics. soft-NJ is not limited to phylogenetics and is suitable for a wide range of continuous gradient-based inference methods on any type of hierarchically structured data. Recent advances in machine learning have also pushed for learning embeddings for hierarchical data such as in natural language processing (Nickel and Kiela 2017, Monath *et al.* 2019, Chami *et al.* 2020a). Soft-NJ provides an alternative algorithm to search through the space of these trees in a differentiable manner.

The approach developed in this work sets a precedent for adopting faster tree reconstruction methods for decoding tree embeddings. Soft versions of algorithms like UPGMA, rapid-NJ, DecentTree (Wang *et al.* 2023) and ninja (Wheeler 2009) used in phylogenetic may offer computation speed and improved. On top of these, there are a host of tree construction algorithms that may offer benefits for inference (Monath *et al.* 2019, Chami *et al.* 2020a). Providing a path and well-tested codebase for these advances is a significant step towards faster phylogenetic optimization.

## 2 Methods

In this section, we provide the necessary background for our proposed phylogenetic embedding technique. First, we recap how phylogenetic models are used for tree inference in maximum likelihood and Bayesian approaches, in particular, variational Bayesian inference. We then introduce hyperbolic space and how phylogenies can be embedded in this space.

### 2.1 Phylogenetic inference

Phylogenetic models compute the likelihood of an aligned set of genetic sequences *D*, which are observed at the tips given a bifurcating tree *T* (Felsenstein 1973). Let $T=T(\tau ,\ell_{\tau })$ denote an unrooted bifurcating tree with topology *τ* and continuous branch lengths $\ell_{\tau }$. A phylogenetic model (denoted M) is a Markov model between the four nucleotide states A,C,G,T/U along the tree at each site in the alignment (Tavare 1986). It has six substitution rates which sum to one and four equilibrium frequencies which also sum to one. We use the GTR model and a simplified version of it called JC69 (Jukes and Cantor 1969) to compute the likelihood of the alignment data *D* given a tree p(D|T,M).

### 2.2 Bayesian phylogenetic models

Bayesian phylogenetics includes prior knowledge of each parameter and seeks the posterior distribution over phylogenetic trees given a multiple sequence alignment. The posterior is p(T,M|D)∝p(D|T,M)p(T)p(M), with, in general, an unknown normalizing constant.

We specify the prior probability of an unrooted tree *p*(*T*) using a Gamma-Dirichlet model (Rannala *et al.* 2012). The Gamma-Dirichlet prior invokes a Gamma distribution (shape 1, rate 0.1) over the total tree length before dividing this length into the branches with an equally weighted Dirichlet distribution (Rannala *et al.* 2012). The GTR model’s prior p(M) is a flat Dirichlet for the six substitution rates and a flat Dirichlet on the four equilibrium frequencies.

### 2.3 Variational inference

VI minimizes some measure of divergence between an approximating function *q* from a family of distributions q∈Q and the posterior target p(T,M|D). We use the standard KL-divergence between the two distributions, which after dropping the M and putting it in log space is:

$$\begin{array}{c} \text{KL}(q(T)||p(T|D)=E[\text{log}\;q(T)]-E[\text{log}\;p(T|D)]\\ =E[\text{log}\;q(T)]-E[\text{log}(p(D|T))+\text{log}(p(D))] \end{array}$$

where the expectations are taken with respect to *q*(*T*). The marginal likelihood of the data log p(D) is intractable to compute, however, since the data is constant, we can simply drop this term and optimize to the same optimum. As a result, the so-called evidence lower bound (ELBO) becomes the objective to maximize:

$$L_{\text{ELBO}}=E[\text{log}\;p(T,D)]-E[\text{log}\;q(T)]$$

Maximizing the ELBO is equivalent to minimizing the KL-divergence between the target p(T|D) and variational distributions *q*(*T*) for any given dataset.

### 2.4 Improved VI

The chosen variational distribution *q*(*T*) may be too simple to capture the true posterior distribution, so to allow for more expressive variational distributions, they can be *boosted* with a mixture model. Boosting is the process of attaining stratified samples over multiple variational distributions $q_{k}(T)$ each with weight $\alpha_{k},\;k\in 1,2,\ldots ,K$. Each sample can be computed with *M* importance samples as done in the stratified importance weighted auto-encoder (SIWAE) (Morningstar *et al.* 2021):

$$L_{\text{SIWAE}}=E_{q}\left[\text{log}\;\frac{1}{M}\sum_{m=1}^{M}\sum_{k=1}^{K}\alpha_{k}\frac{p(T,D)}{q_{k}(T)}\right]$$

Compared to other objectives, this version of the ELBO has improved expressivity and encourages the mixtures not to collapse onto each other (Burda *et al.* 2016, Morningstar *et al.* 2021). We optimize the parameters of the variational distribution to maximize the SIWAE.

Unless otherwise stated, we selected the hyper-parameters *M *=* *1 importance samples, *K *=* *3 boosts (mixtures) with equal initial weights $\alpha_{k}=1/K$. We use PyTorch’s Adam optimizer with a learning rate of 0.1. The learning rate decayed according to ${\left(t+1\right)}^{-0.5}$, where *t* is the iteration number.

### 2.5 Hyperbolic space

**Figure 1.**
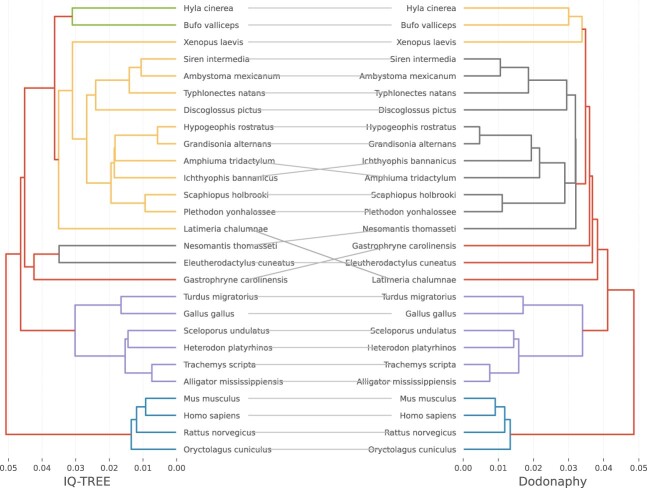
Maximum likelihood tree found by IQ-TREE compared to Dodonaphy for dataset 1.

We model *d*-dimensional hyperbolic space by a hyperboloid $H^{d}=\{u\in R^{d+1}:\langle u,u\rangle =-1\}$, where the Lorentz inner product is $\langle u,v\rangle =-u_{0}v_{0}+u_{1}v_{1}+\cdots +u_{d}v_{d}$. This is a sheet sitting in the ambient space $R^{d+1}$, as illustrated in Fig. 1 of our earlier work (Macaulay *et al.* 2023). The distance between two points on the sheet is

(1) $$d_{\kappa }(u,v)=\frac{1}{\sqrt{-\kappa }}\text{arcosh}(-\langle u,v\rangle ),$$

where κ<0 is the curvature of the manifold. Based on previous work, we select three dimensions *d *=* *3 (Macaulay *et al.* 2023).

### 2.6 Encoding trees in $H^{d}$

To initialize an embedding in hyperbolic space, we take a tip–tip distance matrix from a given phylogenetic tree: *D_T_*. Dodonaphy then uses Hydra+ to embed each taxon with a location ${\vec{z}}_{i}$ in hyperbolic space with *d* dimensions ${\vec{z}}_{i}\in H^{d}$. Hydra+ is a recent adaption of multi-dimensional scaling to hyperbolic space (Keller-Ressel and Nargang 2020). It is an optimization algorithm that minimizes the stress of the embedding, i.e. it minimizes the difference between the given distance matrix *D_T_* and the pairwise distances in hyperbolic space $D_{ij}=d_{\kappa }({\vec{z}}_{i},{\vec{z}}_{j})$. The result is a set of embedding locations in ${\vec{z}}_{i}\in H^{d}$, one for each tip *i* in the phylogenetic tree.

Note that this is an approximate embedding technique, so an encoded tree may not decode back to the originally given tree.

### 2.7 Encoding tree distributions in $H^{d}$

To encode a variational distribution over trees in hyperbolic space, each taxon requires a variational distribution in $H^{d}$. To initialize an embedding, for each taxon, we centred a distribution around the point ${\vec{z}}_{i}$ as in the previous section. We set the covariance to be diagonal, i.e. mean-field, using a coefficient of variation of 20 compared to the smallest tip–tip distance.

Each variational distribution is a multivariate-Normal N(μ,Σ) projected from the tangent space at ${\left(1,0,0,\ldots \right)}^{⊺}$, which is Euclidean space $R^{d}$. Points $z\in R^{d}$ are projected onto the Hyperboloid by modifying the first coordinate:

(2) $$z_{0}\mapsto \sqrt{1+\sum_{i=1}^{d}z_{1}^{2}}$$

and the remaining coordinates $z_{1},\ldots ,z_{d}$ remain the same. The technique is computationally cheap and previously produced similar results to wrapping using an exponential transformation (Nagano *et al.* 2019, Macaulay *et al.* 2023).

### 2.8 Algorithm

We are now set up to describe our algorithm. First, we embed genetic sequences as points (or continuous distributions for VI) in hyperbolic space using Hydra+. Then, we work with the embedded data to optimize the tree (or tree distribution). From a set of embedded points, we compute the neighbour joining tree and compute the cost function *C* (e.g. the phylogenetic likelihood or SIWAE) on that tree. The overall goal is to maximize the cost function by optimizing the embedding parameters (locations or variational distributions).

#### 2.8.1 Differentiable optimization in tree space

We compute the gradient of the cost function *C* with respect to the embedding parameters using automatic differentiation. Automatic differentiation tracks every arithmetic operation in a numerical procedure to provide the analytical derivative of the procedure. From the *n* embedding locations ${\vec{z}}_{i}\in H^{d}$ we compute the pairwise distances *D* using Eq. (1). Then soft-NJ transforms these distances to the neighbour joining tree in the space of trees $T\in T^{n}$. Finally, the tree branch lengths are feed into the loss function $L:T^{n}\to R$ specific to each inference method (e.g. tree likelihood). In summary, this series of transformations is:

(3) $${\left(H^{d}\right)}^{n}\overset{d_{\kappa }}{\to }R^{\left(\begin{array}{c} n\\ 2 \end{array}\right)}\overset{\text{soft-NJ}}{\to }T^{n}\overset{L}{\to }R.$$

The computation of the Jacobian is what guides the optimizer towards uphill, however it is also required in VI to offset the deformations of volume elements in this series of transformations from ${\left(H^{d}\right)}^{n}$ to R. Whilst analytical Jacobains exist to the first (Macaulay *et al.* 2023) and last transform, the impasse is that neighbour joining is not a differentiable algorithm since it selects taxa recursively. By using automatic differentiation and a differentiable version of neighbour joining, we can apply the chain rule through this process to extract the Jacobian as required for VI. Below we present a differentiable version of neighbour joining based on the soft-sort algorithm.

#### 2.8.2 Soft-NJ

From a set of *n* leaf locations ${\left\{u_{i}\right\}}_{i=1}^{n}$ on the hyperboloid, we decode a tree using soft neighbour joining–passing gradients from leaf locations into branch lengths on the tree. Neighbour joining proceeds by recursively connecting the *closest* two taxa according in an adjacency matrix *Q* (Saitou and Nei 1987).

To select this minimum in a differentiable manner, we make use of the soft-sort algorithm (Prillo and Eisenschlos 2020). Soft-sort is a continuous relaxation of the arg-sort operator on a vector with a temperature parameter *η* that controls the degree of approximation and impacts the gradient flow throughout the optimization. A colder temperature, closer to zero, reverts the soft-NJ algorithm back to the discrete (hard) version. Throughout this work, we chose a temperature of $\eta =10^{-5}$ for each dataset.

Algorithm 1. soft-argmin(2D matrix *Q*, temperature *η*) in PyTorch

1: Q_flat_ties←Q.view(−1)

2: P_ties←sort(Q_flat_ties.unsqueeze(0).unsqueeze(−1),η)

3: Q_flat←P_ties[:,−1]×torch.cumsum(P_ties[:,-1],-1)

4: P←sort(−Q_flat.unsqueeze(−1),η)

5: flat_indices←torch.arange(Q.numel())

6: soft_indices←(P[:,−1]×flat_indices).sum((−1,0))

7: soft_row,soft_col←unravel_index(soft_indices,Q.shape)

8: **return**soft_row,soft_col

The implementation of soft-NJ in PyTorch is a naive implementation of neighbour joining, however, the crucial difference is selecting argmin(Q) with a soft version of argmin. To do this, we use soft-sort to create a relaxed permutation matrix of the flattened upper-triangle component $\vec{Q}$ of the *Q* matrix as follows:

$$P=\text{softmax}(\frac{-|\text{sort}(\vec{Q})1^{T}-1{\vec{Q}}^{T}|}{\eta })$$

where 1 is a vector of ones. To extract the arg-min of $\vec{Q}$, we simply multiply by the last column of the permutation matrix *P* by the vector ${\left[1,2,3,\ldots \right]}^{T}$. This leads to a one-hot vector indexing the arg-min of $\vec{Q}$, which is easily unravelled into row and column one-hot vectors to use in neighbour joining. Each of these steps is differentiable and summarized in Algorithm 1, allowing gradients to pass from *Q* into the branch lengths on the decoded tree *T*.

In a small extension to the algorithm, we break any possible ties in *P* by performing soft-sort twice. We break ties differentiably by selecting the first minimum element of $\vec{Q}$ using the cumulative sum function. After obtaining the permutation matrix *P*, we extract its last column denoted *P^l^*. We then apply soft-sort to $P^{l}C$, where *C* is the cumulative sum $C_{i}=\sum_{k=1}^{i}P_{k}^{l}$. This modification ensures that the first minimum element in $P^{*}$ is selected, guaranteeing a well-defined output.

#### 2.8.3 Change of variables Jacobian

In light of Eq. (3), we are sampling trees by changing variables from $H^{d\times n}$ to $T^{n}$. To account for density changes in VI, we must include the determinant of the Jacobian each transformation before $T^{n}$. Recall these transforms are for (a) sampling in $H^{d\times n}$ [which is a projection from Euclidean Space as in Macaulay *et al.* (2023) and Chowdhary and Kolda (2018)], (b) transforming by $d_{\kappa }$ (which has no associated Jacobian), and (c) transforming by soft-NJ. The Jacobian of neighbour joining is analytically non-trivial because of the recursive nature of the algorithm. However, the Jacobian of this series of transformations with soft-NJ is easily computed using automatic differentiation. Since the transformations change dimensionalities, the Jacobian matrix *J* will be non-square and we appeal to the generalized Jacobian $\text{det}{\left(J^{*}{}^{\circ}J\right)}^{1/2}$ (Evans 2018).

### 2.9 Implementation

This algorithm is implemented in Dodonaphy, a software for phylogenetic inference *via* hyperbolic embeddings. It uses several Python packages, notably, PyTorch for automatic differentiation (Paszke *et al.* 2019) and DendroPy for some tree handling (Sukumaran and Holder 2010). Dodonaphy is well-tested and freely available at https://github.com/mattapow/dodonaphy. It has an easy to use command line interface and example input data for analysis.

The second release of Dodonaphy, which focuses on using gradient-based inference is available on Zenodo at: https://doi.org/10.5281/zenodo.8357888. Additionally, the results and figures can be reproduced using the scripts available at: https://github.com/mattapow/vi-fig-scripts and a pipeline is available at https://github.com/4ment/dodonaphy-experiments.

## 3 Results and Discussion

In this section, we will demonstrate the empirical performance of gradient-based tree inference using soft-NJ. We will evaluate its performance for both maximum likelihood and VI.

We have selected eight standard benchmark datasets in phylogenetics taken from Lakner *et al.* (2008) and Whidden *et al.* (2020). These datasets are DNA and RNA multiple sequence alignments with between 27 and 64 tip nodes.

### 3.1 Maximum likelihood optimization

We compared the performance of our proposed hyperbolic embedding technique against two state-of-the-art maximum likelihood phylogenetic programs: IQ-TREE and RAxML-NG.

We initialize an embedding in $H^{3}$ with curvature κ=−100 by embedding the BioNJ tree distances Gascuel (1997). We did this by following the hyperbolic multi-dimensional scaling approach of Hydra+ Keller-Ressel and Nargang (2020). We then optimize the embedding locations, the curvature, and the parameters of the GTR Markov model for 2000 epochs. At this stage, the computational time of Dodonaphy is not competitive with state-of-the-art, largely due to the computational overhead of automatic differentiation.

Figure 1 compares the final tree found for DS1 to IQ-TREE. Although the resulting tree is generally similar to IQ-TREE, there are notable differences. Both the topology and, on close inspection, branch lengths are slightly different. It is possible that the continuous parameters are not fully optimized by Dodonaphy because it is simultaneously dealing with optimizing over tree topologies in the embedding space. To address this, we propose a hybrid approach called Dodonaphy+ where we take the tree that Dodonaphy produces and optimize its continuous parameters using the BFGS optimizer available in IQ-TREE.

**Figure 2.**
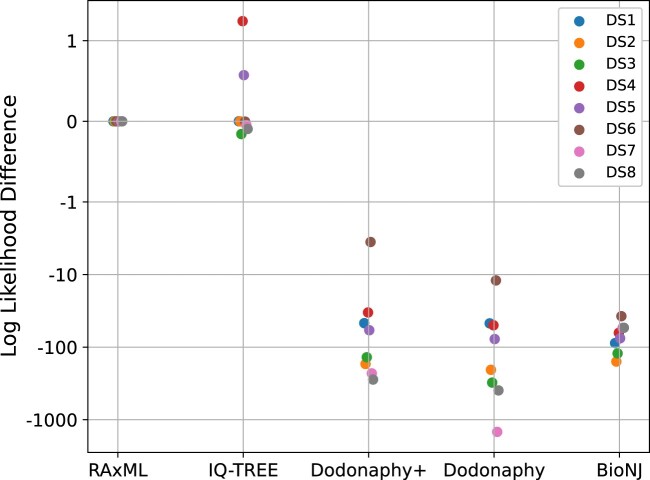
Difference in maximum log-likelihood estimates compared to RAxML across all datasets DS1–8. The vertical axis is negative logarithmic below –1 and linear above it.

To summarize these differences for all datasets we present the log-likelihood under the model in Fig. 2. Dodonaphy outperformed BioNJ for a number of cases demonstrating Dodonaphy’s ability to improve the likelihood. Note that the (negative) log-scale on the vertical axis downplays the significantly poorer performance on DS7. Dodonaphy+ improves the maximum likelihood compared to the original Dodonaphy to varying degrees. In DS5, the improvement is slight but the change is significant for DS7.

We note that after setting the curvature at κ=−100, the final curvatures across all datasets ranged from −58.28 (DS1) to −75.6 (DS3). Previous works have quantified the tree-like of phylogenetic data (Holland *et al.* 2002) as well as the relationship between curvature and the error on the four-point condition (Wilson 2021). These values all fall in the acceptable range previously found on these datasets (Macaulay *et al.* 2023). Allowing the curvature to freely change in the optimization process avoids imposing an arbitrary value.

### 3.2 Local optima by geometric frustration

In practice, the state-of-the-art methods still attain better maximum likelihood estimates, indicating that Dodonaphy’s optimization attains a non-global optimum. Global optimization is generally an unsolved problem and is the subject of significant research. In this case however, some local optima have a geometric interpretation as geometrically frustrated as the entire configuration of embedding points becomes stuck in a locally optimal configuration.

One way to escape such optima is to find different configurations that decode to the same tree. This is the pre-image of a given tree under neighbour joining from the embedding space, backwards through the first two transforms in Eq. (3). For example, swapping the locations of two cherries could decode to the same tree. The appeal of such alterations to the embedding is the altered tree neighbourhood after rearrangement. We speculate that finding embeddings in the same pre-image may provide a way out of the local optima to continue towards the global optimum.

### 3.3 Variational Bayesian inference

Next, we use embedded distributions of trees to perform VI over the space of phylogenies. We take the tip–tip distances from the IQ-TREE and embed each taxon using Hydra+. We then associate each taxon location with a variational distribution centred at this point. The distributions are multivariate-Normals in the tangent space of the origin projected by Eq. (2). We optimize the parameters of these variational distributions and use a JC69 model of evolution to minimize the SIWAE with *K *=* *3 mixtures and *M *=* *3 samples per epoch. After optimizing the SIWAE for 2000 epochs, we drew 10^4^ tree samples from the final variational distribution.

#### 3.3.1 Parameter estimation

We compared our results to the state-of-the-art Metropolis Coupled Markov Chain Monte Carlo (MC^3^) phylogenetic software MrBayes (Ronquist and Huelsenbeck 2003). We ran MrBayes with one cold chain and three heated chains for 10^7^ iterations. We sampled 10^4^ trees evenly throughout this run as an approximation of the posterior and discarded the first 10%. We use the same prior and likelihood models as in MrBayes for a fair comparison between posterior probabilities.

**Figure 3.**
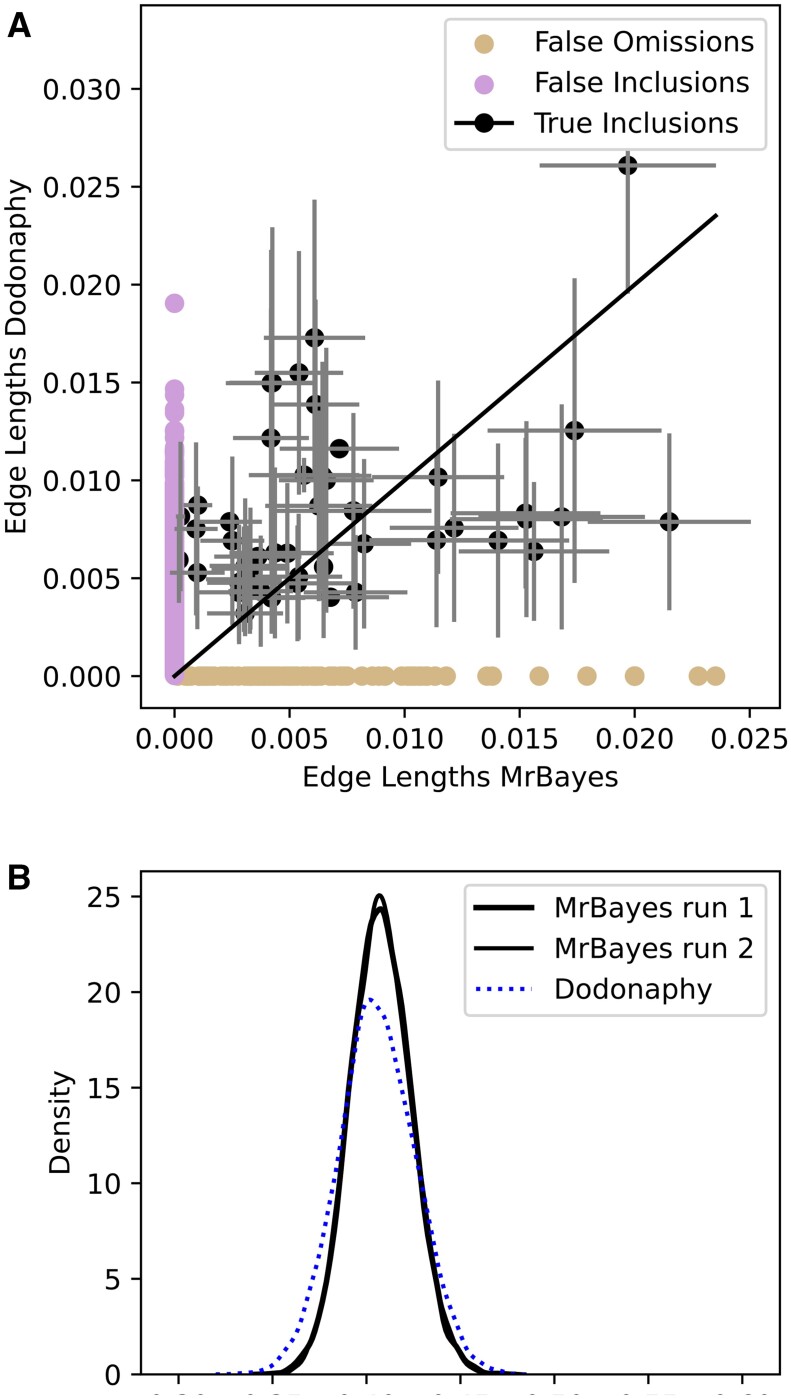
Variational approximation in $H^{3}$ compared to MCMC. Comparison of the split lengths (top), showing true inclusions, false omissions (on horizontal axis) and flase inclusions (on vertical axis). Marker opacity is set by the frequency of the split in MrBayes’ estimate of the posterior. Total tree length (kernel density) estimates (bottom) in the final samples.

The results show moderate agreement between the branch lengths of the posterior, Fig. 3. The estimated split frequencies and total tree lengths compare reasonably to MrBayes when considering the standard errors shown. An exact match is not expected since VI is an approximating algorithm. The support of the inferred tree length closely resembles that of MrBayes, although it is slightly more diffuse with some differences in the recovered topologies.

#### 3.3.2 Performance evaluation

We evaluated the performance of Dodonaphy in comparison to several state-of-art inference techniques in variational Bayesian phylogenetics. We build on a summary of the results recently compiled in Mimori and Hamada (2024) on the same eight datasets. For this section, we used the same model of evolution (Jukes and Cantor 1969) and prior distribution used in these comparisons. The prior is uniform across tree topologies and exponential Exp(10) in the branch lengths. We initialized Dodonaphy to the maximum likelihood tree from IQ-TREE before running the optimization.

Then, we estimated the marginal likelihood of the data over the phylogenetic parameters *θ* using variational Bayesian importance sampling (Fourment *et al.* 2020): p(D)=∫p(D|θ)p(θ)dθ. This estimator uses the variational distribution as an importance distribution for importance sampling:

$$\hat{p}(D)=\frac{1}{N}\sum_{i=1}^{N}\frac{p(D|{\tilde{\theta }}_{i})p({\tilde{\theta }}_{i})}{q({\tilde{\theta }}_{i})},$$

where $q({\tilde{\theta }}_{i})$ is the variational distribution and ${\tilde{\theta }}_{i}∼q(\tilde{\theta })$. We used *K *=* *1 importance samples and *N *=* *1000 samples from the variational distribution to compute this marginal estimator. Although multi-threading over *K* is embarrassingly parallel.

Table 1 extends work in Mimori and Hamada (2024) to compare Dodonaphy with state-of-the-art VI methods. The results from stepping stone MCMC in MrBayes is also included as a baseline comparison. Note that while VBPI-GNN has excellent results it is given topologies as inputs rather than performing topological inference. Geophy and *ϕ*-CSMC are the current state-of-art implementations performing topological and continuous parameter phylogenetic inference.

**Table 1.** Comparison of marginal log-likelihood estimates.

Dataset	DS1	DS2	DS3	DS4	DS5	DS6	DS7	DS8
MrBayes	−7108.42	−26 367.57	−33 735.44	−13 330.06	−8214.51	−6724.07	−37 332.76	−8649.88
VBPI-GNN	−7108.41	−26 367.73	−33 735.12	−13 329.94	−8214.64	−6724.37	−37 332.04	−8650.65
Geophy LOO(3)+	−7116.09	−26 368.54	−33 735.85	−13 337.42	−8233.89	−6735.90	−37 358.96	−8660.48
*ϕ*-CSMC	−7290.36	−30 568.49	−33 798.06	−13 582.24	−8367.51	−7013.83	–	−9209.18
Dodonaphy	−8042.10	−26 777.43	−34 437.62	−15 070.36	−13 702.8	−9595.49	–	–

Dodonaphy provides poorer estimates of the posterior than competing methods. The suboptimal results could be attributed to the continuous hyperbolic variational approximation and again geometric frustration. Underlying this model is the assumption that trees with similar tip–tip distances share similar posterior likelihoods. This assumption is a heuristic that provides an efficient way to encode tree distributions but may constrain the flexibility of the distribution. These findings are also consistent with a variational distribution that is too simple, calling for a more expressiveness. We explore this by boosting the variational distribution.

### 3.4 Effect of boosting

**Figure 4.**
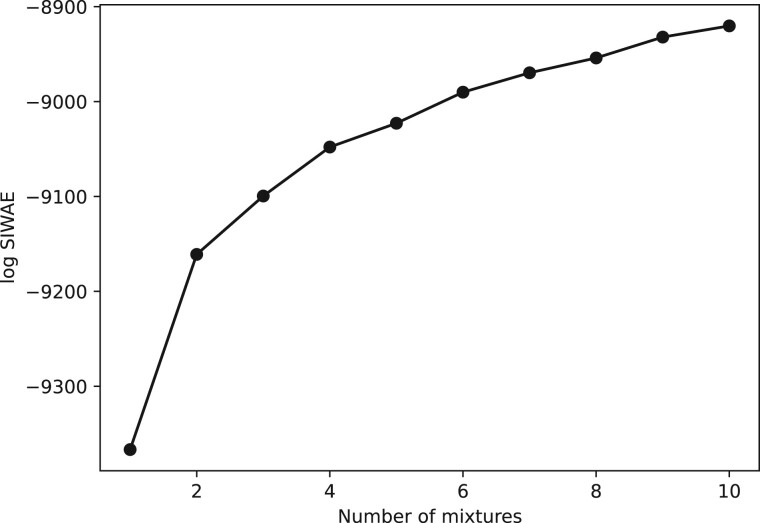
Effect of the number of boosts on the final SIWAE estimate for DS1.

Whilst boosting improves the expressiveness of the variational distribution, it also increases the computational demand of VI by a factor of *K*, so we are interested in the minimal number of mixtures required. To understand the number of boosts required to capture the embedded posterior distribution of trees, we fixed the number of importance samples at *M *=* *3 and varied the number of mixtures *K* from one to ten. We optimized for 2000 epochs starting from the IQ-TREE distances. The final SIWAE value suggests that the presence of additional mixtures improves the variational approximation, although the improvement slowly saturates after *K *=* *3, Fig. 4. Having this flexible variational family increases the inference accuracy and opens up more complex tree distributions.

## 4 Conclusions

Hyperbolic tree embeddings, through the use of soft-NJ, provide a differentiable way to efficiently encode trees and even distributions of trees. Using Dodonaphy, we demonstrated two applications of soft-NJ in maximum likelihood and VI. Whereas, classical phylogenetic methods, both ML and Bayesian, rely on discrete tree operations such as nearest neighbour interchange, Dodonaphy can transcend many of these moves at once. This complements their ability, offering new ways of tree searching, and could be incorporated into existing phylogenetic programs. It also opens up soft versions of tree forming algorithms like rapid-NJ, bio-NJ, ninja, or UPGMA. Additionally, soft alignment algorithms can now integrate with soft tree reconstruction allowing an end-to-end pipeline (Petti *et al.* 2022).

The challenges of non-convex optimization are long-standing, although here, by using embedded point configurations, not single points, we may alleviate this geometric frustration by re-embedding under the pre-image of the decoded tree. Additionally, alternative approximations, full-rank methods and normalizing flows are next steps towards quality variational approximations, allowing flexible and expressive distributions to fully realize the potential of hyperbolic tree optimization.
